# The Application of Vacuum-Assisted Closure Device in the Management of Empyema Necessitans

**DOI:** 10.1155/2016/6805736

**Published:** 2016-08-31

**Authors:** Yasser Aljehani, Zahra Al-Matar, Samah Nawar

**Affiliations:** Department of Surgery, Thoracic Surgery Division, King Fahad Hospital of the University, University of Dammam, P.O. Box 1982, Al-Khobar, Dammam 31441, Saudi Arabia

## Abstract

Vacuum-assisted closure (VAC) is gaining popularity in the management of many types of acute and chronic wounds. The use of VAC devices in thoracic surgery is limited, but it appears to be promising in complex cases of empyema thoraces. We report a case of empyema necessitans, in which VAC was used to achieve complete wound healing after open drainage which was communicating with the pleural space.

## 1. Introduction

Vacuum-assisted closure (VAC) therapy is a well-known treatment modality used in the management of acute and chronic wounds that are difficult to treat [1]. The intrapleural use of VAC in the management of empyema was reported for the first time in 2006 [2]. Recent studies have also shown that it is a safe and efficient adjuvant in the management of severe intrathoracic infections associated with lung resection, mediastinitis related to esophageal leakages, or necrotizing pleuropulmonary infections. The intrathoracic use of VAC in these infections improves residual lung reexpansion and promotes pleural space obliteration [3].

## 2. Case Report

A 27-year-old Saudi female with no significant medical history presented with a picture of parapneumonic pleural effusion. Approximately two weeks prior to this presentation, the patient underwent a laparoscopic drainage of postappendectomy intra-abdominal abscess. The patient was managed in another hospital with a thoracostomy tube for drainage and systemic antibiotics and was discharged after a hospital stay of two months. The patient continued to have a persistent fever.

**Figure 1:**
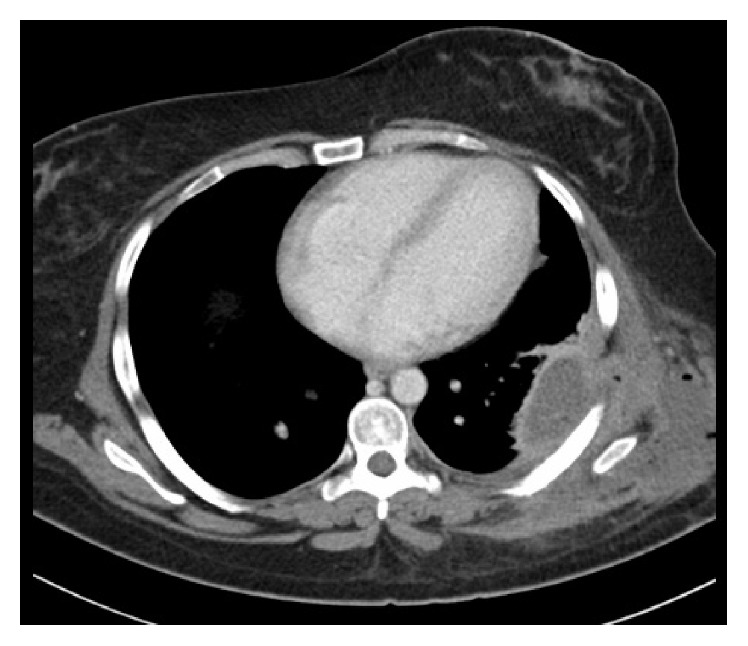
Axial CT scan of the chest demonstrating the intrapleural collection with pockets of air consistent with empyema.

**Figure 2:**
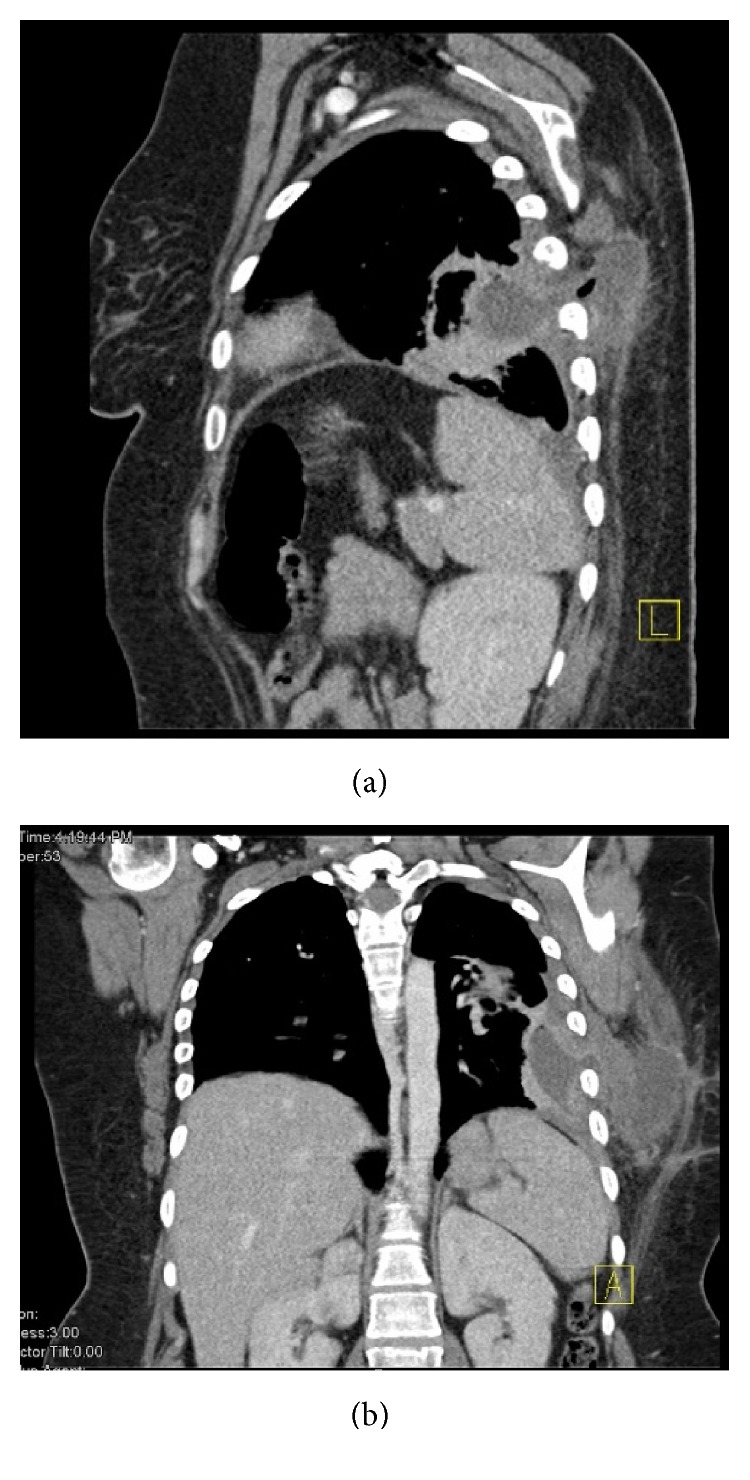
(a) Sagittal reconstruction of CT scan demonstrating the extension of the intrapleural collection into the subcutaneous tissue. (b) Coronal reconstruction demonstrating the same findings consistent with empyema necessitans.

One week after discharge, she presented to our hospital as a case of empyema. She underwent thoracotomy and decortication and antimicrobial therapy directed against MDR* Pseudomonas*. Around three weeks postoperatively, she developed a lung abscess for which a pigtail catheter was inserted under interventional radiology guidance. She improved dramatically and was discharged. One week later, the patient started to complain of fever, mild productive cough, and left-sided pleuritic chest pain. On initial examination, the patient was tachycardic with a low-grade fever of 37.9°C. There was a fluctuant, tender, erythematous mass around the previous thoracotomy wound as well as diminished breath sounds over the left hemithorax. The laboratory data showed high ESR (120); however, there was no marked leukocytosis. The chest X-ray showed opacity on the left lower zones. The computed tomographic (CT) scan of the thorax showed intrapleural loculated collection communicating and extending to the subcutaneous tissue (Figure 1). The radiological picture is consistent with empyema necessitans (Figures 2(a) and 2(b)). Decortication and debridement of the purulent and subcutaneous tissues were undertaken, leaving a gap and a large communication between the pleural space and subcutaneous tissue due to the extensive debridement. VAC (KCL, San Antonio, TX) was used for wound closure. The patient recovered from the surgery uneventfully. Acid Fast Bacilli were ruled out and culture of the purulent fluid obtained from plural space grew* Bacteroides*. The patient was placed on intravenous antibiotic guided by sensitivities. The VAC device was changed on the 3rd postplacement day and then was frequently changed every 3rd day, initially under general anesthesia and subsequently under sedation. The VAC was set at a continuous suction mode of not more than 100 mmHg. Cultures were taken regularly and, on the 18th day of admission, tests showed negative growth. The patient's condition improved clinically, as evidenced by her laboratory tests. Repeated CT scans showed resolution of all collections, both pleural and subcutaneous. Her follow-up at six months and one year showed complete resolution of her condition.

## 3. Discussion

Empyema is defined as collection of purulent fluid in the pleural space, commonly caused by pneumonia as sequel of parapneumonic effusion. The pus collection can sometimes dissect through the intercostal space reaching the subcutaneous tissue and presents as an abscess or as an asymptomatic mass, commonly on the anterolateral chest, a condition that is known as empyema necessitans.* Mycobacterium tuberculosis* is the most common pathogen; less common etiological agents include* Actinomyces*,* Streptococcus pneumonia*, and* Staphylococcus aureus* [4]. Our case demonstrated the growth of* Bacteroides*. The initial treatment modality for early stage empyema includes thoracostomy tube drainage along with antimicrobial therapy [5]. More invasive techniques such as thoracoscopy (VATs) or thoracotomy with decortication are usually required for advanced stages of empyema. If these measures fail, open windows such as Eloesser flap or Clagett window are indicated in medically unstable patients [5]. Although open windows are safe and efficient techniques for the management of pleural infections, they require prolonged hospitalization and daily changes of the intracavitary wound dressings [6].

In 2006, Varker and Ng described the successful use of VAC system on a patient with postlobectomy empyema, the result of which showed complete healing of the wound with minimal change in the chest contour and no complications [2]. Since then other reports have also described the successful use of intrathoracic VAC system [7–9]. VAC devices have been used in the management of postpneumonectomy empyema with bronchopleural fistula. An experience by Han and Kim demonstrated not only success but also reduction of cost and proper utilization of resources compared to the standard approach of delayed closure known as Clagett window [10]. The safety of direct application of VAC on lung parenchyma has been less frequently reported [2, 7, 8]. In this case, VAC was used for wound closure over the ribs with direct communication with the intrapleural space.

The negative pressure applied by the VAC accelerates wound healing as it enhances the blood flow in the treated area and promotes healthy granulation tissue growth. Moreover, it decreases edema and excessive fluid from the wound and limits bacterial colonization [1]. The intrathoracic application of the VAC system may result in a shorter period of hospitalization as this treatment can be provided on an outpatient basis [2, 8]. Reports have shown that patients can have a complete recovery following the use of VAC in the thorax [8, 9].

In our patient, once the signs and symptoms of infection had resolved, the patient was discharged with the VAC device to be followed up as an outpatient. In our setting, 21 days were required to remove the device. No air leak or bleeding from lung parenchyma was observed and spontaneous closure of the wound site was achieved without a need for further intervention.

## 4. Conclusion

VAC has been proven to be successful in the treatment of thoracic infections, including empyema necessitans, and it minimized the need for more invasive procedures. Furthermore, VAC was more convenient for the patient and reduced the economic burden on the health institute due to shorter length of hospitalization. We advocate this treatment modality and we recommend randomized trials to further evaluate its efficacy.
